# International dietary quality index and its association with diabetes in RaNCD cohort study

**DOI:** 10.1038/s41598-024-77165-4

**Published:** 2024-10-24

**Authors:** Zahra Mokhtari, Hadi Abdollahzad, Shahab Rezaeian, Neda Izadi, Mitra Darbandi, Farid Najafi, Yahya Pasdar

**Affiliations:** 1 Student Research Committee, Kermanshah University of Medical Sciences, Kermanshah, Iran; 2 Department of Nutrition, School of Medicine, Urmia University of Medical Sciences, Urmia, Iran; 3 Infectious Diseases Research Center, Health Institute, Kermanshah University of Medical Sciences, Kermanshah, Iran; 4 Research Center for Social Determinants of Health, Research Institute for Endocrine Sciences, Shahid Beheshti University of Medical Sciences, Tehran, Iran; 5 Research Center for Environmental Determinants of Health (RCEDH), Health Institute, Kermanshah University of Medical Sciences, Kermanshah, Iran; 6 Nutritional Sciences Department, School of Nutritional Sciences and Food Technology, Isar Square, Tehran, Iran

**Keywords:** Diabetes, Dietary quality index, DQI-I, Diet, Persian cohort, Endocrinology, Nutrition

## Abstract

Diabetes and its complications pose a significant threat to global health. Various factors contribute to the development of diabetes, with diet being an important trigger. The Dietary Quality Index-International (DQI-I) serves as an indicator of changes in diet and its association with chronic diseases, including diabetes. The aim of this study is to examine the association between DQI-I and diabetes in adults. Data from the first phase of the Ravansar Non-Communicable Disease Cohort Study (RaNCD) were used for this cross-sectional study. The study included individuals from western Iran aged between 35 and 65 years. The DQI-I was used to assess diet quality and the essential aspects of a healthy diet. Multiple logistic regression analyses were performed to compare DQI-I total score and diabetes. A total of 7,079 individuals were included, including 608 diabetic and 6,471 healthy individuals. The mean DQI-I score was 60.51 ± 8.47 in healthy individuals and 63.12 ± 8.64 in diabetics. The odds of developing diabetes were higher in individuals with a higher DQI-I (adjusted odds ratio: 1.49, 95% CI: 1.30–1.73). The variety was 13.43 ± 4.47 in diabetics and 12.59 ± 4.79 in healthy individuals. Adequacy was 33.23 ± 3.71 in diabetics and 33.79 ± 3.37 in healthy individuals. Moderation was 13.27 ± 6.05 in diabetics and 11.79 ± 5.47 in healthy individuals. The overall balance was 2.88 ± 2.21 in the healthy group and 2.61 ± 2.13 in the diabetics. The macronutrient ratio was 2.15 ± 1.88 in the healthy group and 2.04 ± 1.84 in the diabetics. The fatty acid ratio was 0.72 ± 1.29 in the healthy group and 0.56 ± 1.17 in the diabetic group. The overall balance score was higher in the healthy subjects. The DQI-I total score was higher in diabetics, indicating a positive association between diabetes and the DQI-I. Therefore, the importance of continuous dietary management and education of diabetic patients should be emphasized.

## Introduction

Diabetes and its complications pose a significant threat to global health. It is the fourth leading cause of death worldwide, claiming 1.5 million lives annually^1,2^. According to the International Diabetes Federation (IDF), the worldwide prevalence of diabetes is estimated at 425 million, with 279 million residing in urban areas and 146 million in rural areas. The IDF predicts that the prevalence of diabetes in people aged 20 to 79 years will increase to 12.2% (2.783 million) by 2045^1,3^. In Iran, the prevalence of diabetes in people aged 18 years and older is estimated at 14.15%, with a further 24.79% expected to have prediabetes in 2021. These figures represent a 45.5% increase in diabetes prevalence compared to 2016^4^.

Lifestyle changes, including a healthy diet, regular physical activity, not smoking and moderate alcohol consumption, can delay or even prevent the development of diabetes^5–8^. Dietary changes have a significant impact on blood glucose control and play a crucial role in the management of diabetes^9^. A cross-sectional study conducted on 646 participants shows that nutritional status is negatively associated with some components of health-related quality of life in diabetic patients, and that dietary intervention can reduce the negative impact of diabetes on health-related quality of life^9^.

The Dietary Quality Index-International (DQI-I) is an index that evaluates changes in diet and their association with metabolic factors^10^. A notable advantage of this index is that it takes into account both factors of inadequate food consumption (such as variety and sufficiency) and factors of excessive food consumption (such as moderation and balance). Unlike many other food quality indicators, the DQI-I addresses health problems related to overeating^11^. Many food quality indicators focus primarily on malnutrition or deficiencies, which are a major problem, especially in certain population groups. However, as dietary habits have evolved and lifestyles have changed, overeating has become increasingly prevalent, especially in developed countries. As the DQI-I takes into account both insufficient and excessive consumption, it provides a more comprehensive assessment of dietary habits. A cross-sectional study conducted in 2021 found that a high DQI-I score was associated with a lower prevalence of diabetes and lower blood pressure^12^.

Despite the growing impact of diabetes on public health, there is a lack of comprehensive research on diet quality in diabetes. Therefore, the aim of this study is to evaluate the quality of diet in diabetic patients. By identifying the major problematic dietary factors associated with diabetes, the results of this study can guide the development of programs to improve the health of patients and society. This study investigated whether diet quality as measured by the DQI-I is associated with a lower risk of diabetes.

## Methods

### Study population

This cross-sectional study was conducted using data from the first phase of the Ravansar Non-Communicable Disease Cohort Study (RaNCD)^13^. The Ravansar cohort, a branch of the Prospective Epidemiological Research Studies in IrAN (PERSIAN), aims to determine the incidence of non-communicable diseases and their associated outcomes, including hospitalization and mortality. The initial phase of the RaNCD cohort study was conducted in 2014, involving 10,047 participants aged between 35 and 65 years. This study included all participants from the initial phase of the RaNCD cohort. Participants with abnormal daily energy intake (less than 800 or more than 4,200 kcal/day), incomplete data, and chronic diseases such as cancer, kidney failure, chronic lung disease, rheumatic disease and gallstones were excluded from the study population (*n* = 2,968). Finally, data from 7,079 participants at baseline, consisting of 608 individuals with diabetes and 6,471 healthy individuals were included in the study.

### Measurements

The information about the participants was collected in the form of a digital and an online questionnaire by trained experts at the cohort center. Individuals’ demographic data, including age, gender and marital status were collected using standard Persian cohort questionnaires. Principal component analysis (PCA) was used to determine the socioeconomic status (SES) of individuals. Socioeconomic status included employment status, education level, employment history, place of residence, number of domestic and foreign trips, access to landlines and mobile phones, and internet and mobile phone usage.

The standardized physical activity questionnaire of the PERSIAN cohort was used to determine the participants’ level of physical activity. This questionnaire contained 22 questions about the individual’s daily activities. The answers from the questionnaire were then extracted and used to calculate the metabolic equivalent of labor per hour per day (MET/h/day). Then METs were classified as low (24-36.5 MET/h per day), moderate (36.6–44.4 MET/hours per day), and vigorous (≥ 44.5 MET/h per day)^14^.

Body measurements were taken using the InBody 770 body composition device. Weight measurements were taken using the same device with a precision of 100 g. Height was measured using the Biospace Co. Stadiometer 370 automated device, and the recorded heights were then documented in centimeters in the InBody 770 device. The body mass index (BMI) was calculated using the formula weight (kg)/height^2^ (m).

Blood pressure measurements followed the standard methodology of the Persian cohort. To perform biochemical tests such as lipid profile, blood glucose and liver enzymes, blood samples were collected using standard vacutainer blood collection techniques, with participants assuming a seated position and a tourniquet applied. Further details have already been published in the RaNCD cohort study protocol^13^.

Diabetes was identified by a fasting blood sugar (FBS) ≥ 126 mg/dl dL [7 mmol per L] and/or use of diabetes medications (insulin and/or oral hypoglycemic agents) in individuals at baseline in the cohort study^15^.

### Nutritional assessment

Nutritional status was assessed using the Iranian National Food Frequency Questionnaire (FFQ)^16^, a comprehensive tool for assessing dietary habits. The FFQ asks about the consumption of various foods and beverages in the past year. The questionnaire consists of 118 food items that are specifically relevant to the Iranian population. It includes a variety of categories such as bread, grains, cereals, meat and meat products, milk and dairy products, vegetables, fruits, oils and oilseeds, sugar, various foods, spices and dietary supplements. In addition, the questionnaire takes local foods into account to ensure cultural relevance. The amount of consumption of each food item was converted to grams per day (g/day) using Nutrition IV software to measure the daily intake of food. In this study, the FFQ was used to calculate an index score representing the overall diet quality of the participants.

### Dietary quality index-international (DQI-I)

To determine the dietary quality index-international score for each person, the data from the FFQ questionnaire, which contains 118 foods specific to that person, was used. The overall structure and scoring system of the DQI-I is based on the study conducted by Kim et al.^10^. The index comprises four main categories, with variety assessed in two subgroups: Overall variety and variety in protein sources. To assess overall variety, the maximum score was awarded if at least one meal per day contained products from all five food groups (meat, poultry, fish, eggs; dairy; beans/grains; fruits and vegetables). If one of these food groups was not consumed, 3 out of a total of 15 points were deducted for each missing group. For the variety of protein sources, a score of 5 was awarded if the intake came from three different protein sources per day. The score was reduced to 3, 1 and 0 points if the number of different sources fell to 2, 1 and 0 respectively. In the adequacy subgroup, eight components were assessed and points were awarded based on the percentage of recommended intake achieved, ranging from 0 points for no consumption (0%) to 5 points for complete consumption (100%). The adequacy of fruit, vegetable, grain, and fiber intake depended on energy intake, while protein intake was considered adequate if it accounted for at least 10% of total energy intake. The recommended intake for iron, calcium and vitamin C were determined based on the Dietary Reference Intake (DRI), which varies according to age and gender. Within the “moderation” subgroup, nutrient intake (total fat, saturated fat, cholesterol and sodium) was categorized into three levels based on their impact on health. The lowest level received the highest score of 6 points, the highest level received the lowest score of 0 points, and the average level received a score of 3 points. Another aspect of the DQI-I was the evaluation of “empty- calorie foods”, which have a low nutrient density. If the energy derived from such foods accounted for more than 10% of the total energy intake per day, the lowest score was assigned, while consumption of less than 3% received the highest score. Examples of these foods are ice cream and cookies, which consist mainly of carbohydrates and fats and contain fewer vitamins, minerals and amino acids^17^. In the final category, the overall balance, the diet was assessed in terms of the proportion of energy sources and the composition of fatty acids. Each component was awarded 6 or 4 points. The scores of the individual subgroups within the four main categories were added together to give an overall DQI-I score ranging from 0 to 100, with 0 being the lowest score and 100 being the highest possible score.

### Statistical analysis

The statistical analyses were conducted using STATA version 17 software. Descriptive statistics were used to present the quantitative variables, including mean and standard deviation, while the qualitative variables were described with frequency and percentage. The t-test was used to compare the quantitative variables between the two groups studied, i.e. individuals with diabetes and healthy individuals. In addition, the chi-square test was used to assess the qualitative variables between the groups. In addition, the chi-square test was used to determine the distribution of categorical variables and DQI-I scores between individuals. Analysis of variance (ANOVA) was used to evaluate the differences of continuous variables in the tertiles of the DQI-I. Binary logistic regression analysis was used in both crude and adjusted models to examine the association between dietary quality index and diabetes (age, gender, smoking and alcohol in model 1; additionally,  marital status, education, employment and socioeconomic status in model 2).

## Results

The total number of subjects in this study was 7,097, with an average age of (47.16 ± 8.31 years). In the general population, 44.38% were men and 55.62% were women. A total of 608 diabetics participated in the study. The average age of the diabetics was 51.64 years and that of the healthy subjects was 46.73 years, which means that the diabetics were older than the healthy subjects. Of the diabetics, 42.76% were male and 57.24% female. In the healthy group, 44.54% were male and 55.46% were female. It was found that the majority of diabetics lived in urban areas and had a higher SES than the healthy individuals. Of note, the diabetics were less intensely physically active, and this difference was statistically significant (*P* < 0.001) (Table 1).

**Table 1:** Characteristics of participants by diabetes status.

Variable		All subjects	Healthy subjects	Diabetic subjects	*P*-value
Number	–	7079	6471	608	-
Age	Year	47.16 ± 8.31	46.73 ± 8.27	51.64 ± 7.41	< 0.001
Gender	Men	3142 (44.38)	2882 (44.54)	260 (42.76)	0.400
	Women	3937 (55.62)	3589 (55.46)	348 (57.24)	
Marital status	Single	333 (4.70)	325 (5.02)	8 (1.32)	< 0.001
	Married	6332 (89.45)	5772 (89.20)	560 (92.11)	
	Other	414 (6.74)	374 (5.78)	40 (6.58)	
Occupation	Workless	114 (1.61)	98 (1.51)	16 (2.63)	< 0.001
	Practitioner	3211 (45.36)	2974 (45.96)	237 (38.98)	
	Housekeeper	141 (1.99)	118 (1.82)	23 (3.78)	
	Other	608 (51.04)	3281 (50.70)	332 (54.61)	
Residence area	City	4084 (57.69)	3696 (57.1)	388 (63.82)	0.001
	Rural	2995 (42.31)	2775 (42.88)	220 (36.18)	
Socioeconomic status	Poor	2993 (41.51)	2683 (41.48)	245 (41.48)	0.016
	Middle	1402 (19.81)	1281 (19.80)	121 (19.93)	
	Rich	2737 (37.68)	2505 (38.72)	233 (38.22)	
Smoking status	No	2948 (69.02)	2707 (69.54)	241 (63.76)	0.100
	Current	451 (10.56)	405 (10.40)	241 (12.17)	
	Former	254 (5.95)	231 (5.93)	23 (6.08)	
	Passive	618 (14.47)	550 (14.13)	68 (17.99)	
Alcohol consumption	No	6775 (95.71)	6189 (95.64)	586 (96.38)	0.390
	Yes	304 (4.29)	282 (4.36)	22 (3.62)	
Physical activity	Low	2098 (29.64)	1891 (29.22)	207 (34.05)	< 0.001
	Moderate	34.65 (48.95)	3157 (48.79)	308 (20.66)	
	Vigorous	1516 (21.42)	1423 (21.99)	93 (15.30)	

Variables are reported as (frequency (%)) and p-values are obtained from the chi-square test. The age is reported as (mean ± standard deviation) and p-value is obtained from the t-test.

Table 2 shows the anthropometric, biochemical and nutritional characteristics of the participants. Anthropometric indicators were higher in diabetics than in healthy individuals. However, these differences were not statistically significant for muscle mass (*P* = 0.629), fat-free mass (FFM) (*P* = 0.941) and lean body mass (LBM) (*P* = 0.9213). The biochemical indicators showed that the diabetic subjects had higher values compared to the healthy subjects, and these differences were statistically significant (*P* < 0.05).

**Table 2:** Anthropometric, biochemical and nutritional characteristics by diabetes status.

Variable	All subjects	Healthy subjects	Diabetic subjects	*P*-value
Weight (kg)	72.25 ± 13.49	71.99 ± 13.54	75.04 ± 12.62	< 0.001
Body mass index (kg/m^2^)	27.41 ± 4.65	27.28 ± 4.66	28.82 ± 4.33	< 0.001
Waist circumference (cm)	97.06 ± 10.59	96.73 ± 10.61	100.57 ± 9.75	< 0.001
Body fat percentage	34.05 ± 9.64	33.81 ± 9.51	36.65 ± 8.55	< 0.001
Waist to hip ratio	0.94 ± 0.06	0.93 ± 0.06	0.95 ± 0.06	< 0.001
Skeletal muscle mass (kg)	26.08 ± 5.61	26.09 ± 5.65	25.98 ± 5.08	0.629
Fat free mass (kg)	47.22 ± 9.72	47.22 ± 9.34	47.19 ± 8.44	0.941
Lean muscle mass (kg)	44.65 ± 8.78	44.65 ± 8.85	44.62 ± 7.99	0.921
Body fat mass (kg)	25.03 ± 9.55	24.77 ± 9.54	27.84 ± 9.16	< 0.001
Systolic blood pressure (mmHg)	107.67 ± 16.76	106.93 ± 16.41	115.52 ± 18.40	< 0.001
Diastolic blood pressure (mmHg)	69.45 ± 9.79	69.16 ± 9.64	72.55 ± 10.79	< 0.001
Total cholesterol (mg/dl)	185.49 ± 38.07	184.99 ± 37.38	190.81v44.67	< 0.001
Triglyceride (mg/dl)	136.54 ± 81.83	132.45 ± 75.82	180.07v121.39	< 0.001
HDL cholesterol (mg/dl)	46.62v11.30	46.82 ± 11.28	44.54v11.26	< 0.001
LDL cholesterol (mg/dl)	102.06 ± 25.45	104.15 ± 28.36	101.86 ± 25.15	0.030
Fasting blood sugar (mg/dl)	97.18 ± 30.19	90.40 ± 9.72	169.35 ± 62.58	< 0.001
Sleep time (h)	7.12 ± 1.20	7.13 ± 1.20	7.01v1.28	0.020
Energy (Kcal/day)	24.65.26 ± 736.98	2506.77 ± 733.87	2372.76 ± 759.20	< 0.001
Saturated fatty acid (g/day)	29.92 ± 15.77	29.97 ± 15.79	29.64 ± 15.55	0.453
Monounsaturated fat (g/day)	16.24 ± 8.44	16.38 ± 8.49	14.84 ± 7.82	< 0.001
Polyunsaturated fat (g/day)	8.90 ± 4.45	8.92 ± 4.43	8.63 ± 4.60	0.104
Fiber (g/day)	47.19 ± 20.53	46.93 ± 20.42	50 ± 21.43	< 0.001
Iron (mg/day)	23.96 ± 9.61	23.89 ± 9.53	24.77 ± 10.34	0.030
Calcium (mg/day)	732.89 ± 389.41	727.74 ± 387.39	787.68 ± 406.66	< 0.001
Vitamin C (mg/day)	253.31 ± 137.95	251.62 ± 137.44	271.25 ± 142.18	< 0.001

*HDL* High-density lipoprotein, *LDL* Low-density lipoprotein, Quantitative variables are reported with (mean ± standard deviation), and p-values for quantitative variables are obtained from the t-test.

With regard to food intake, it was found that healthy individuals had a significantly higher average energy intake than diabetics (2506.77 ± 733.87 vs. 2372 ± 759.20). The average intake of monounsaturated fatty acids (MUFA) was 16.38 ± 8.49 g in the healthy group and 14.84 ± 7.82 g in the diabetics. The average consumption of saturated fatty acid (SFA) was 29.97 ± 15.79 g in the healthy subjects and 29.46 ± 15.5 g in the diabetics. The average consumption of polyunsaturated fatty acids (PUFA) was 8.92 ± 4.43 g in the healthy subjects and 8.63 ± 4.60 g in the diabetics. However, the differences were not statistically significant (SFA (*P* = 0.453) and PUFA (*P* = 0.104)). Dietary fiber content was 50 ± 21.43 g in the diabetic group and 46.93 ± 20.42 g in the healthy group and was statistically significant (*P* < 0.001).

Table 3 shows the comparison of DQI-I scores and its subgroups such as variety, adequacy, moderation and overall balance in healthy individuals and diabetics. The results show that diabetics had a higher overall dietary quality index score compared to healthy individuals (63.12 ± 8.64 vs. 60.51 ± 8.47). In addition, the mean scores for variety were 13.43 ± 4.47 in the diabetic group and 12.59 ± 4.79 in the healthy group. Adequacy was 33.79 ± 3.37 in the diabetic group and 33.23 ± 3.69 in the healthy group. Moderation was also higher in the diabetics than in the healthy subjects (13.27 ± 6.05 vs. 11.79 ± 5.47). Variety, adequacy, and moderation were statistically significant (*P* < 0.001). However, the score for overall balance was 2.88 ± 2.21 in the healthy group and 2.61 ± 2.13 in the diabetics; this difference was also statistically significant (*P* = 0.004).

**Table 3:** DQI-I scores and its subgroups between diabetic and healthy subjects.

Variable	All subjects	Healthy subjects	Diabetic subjects	*P*-value
DQI-I total score	60.74 ± 8.51	60.51 ± 8.47	63.12 ± 8.64	< 0.001
Variety	12.66 ± 4.77	12.59 ± 4.79	13.43 ± 4.47	< 0.001
Adequacy	33.28 ± 3.69	33.23 ± 3.71	33.79 ± 3.37	< 0.001
Moderation	11.92 ± 5.54	11.79 ± 5.47	13.27 ± 6.05	< 0.001
Overall balance	2.86 ± 2.20	2.88 ± 2.21	2.61 ± 2.13	0.004

The total scores of DQI-I (Diet Quality Index-International), variety, adequacy, moderation, and overall balance are presented as (mean ± standard deviation). The p-values are obtained from t-test.

According to the data presented in Table 4, variety component scores were higher in diabetics, although protein variety did not show statistical significance (*P* = 0.143). Similarly, all components of adequacy had higher values in diabetics, but cereals (*P* = 0.791), calcium (*P* = 0.487) and vitamin C (*P* = 0.711) were not statistically significant. In the moderation group, all components were higher in the diabetics, except for cholesterol, which was not statistically significant (*P* = 0.453). However, the saturated fat component was higher in the healthy group and this difference was statistically significant (*P* = 0.046). On the other hand, both components of the overall balance, including the macronutrient ratio, were 2.04 ± 1.84 in the diabetic group and 2.15 ± 1.88 in the healthy group. The fatty acid ratio was 0.56 ± 1.17 in the diabetic group and 0.72 ± 1.29 in the healthy group. Both components of the overall balance were higher in healthy subjects than in diabetics. The value for the macronutrient ratio was not significant (*P* = 0.176) and the value for the fatty acid ratio was significant (*P* = 0.003).

**Table 4:** DQI-I scores subgroups components in healthy subjects and diabetes.

Variable	All subjects	Healthy subjects	Diabetic subjects	*P*-value
Variety	12.66 ± 4.77	12.59 ± 4.79	13.43 ± 4.47	< 0.001
Overall variety	10.61 ± 3.51	10.57 ± 3.53	11.31 ± 3.27	< 0.001
Protein variety	2.03 ± 1.59	2.02 ± 1.60	2.12 ± 1.49	0.143
Adequacy	33.28 ± 3.69	33.23 ± 3.71	33.79 ± 3.37	< 0.001
Vegetable	4.35 ± 121	4.34 ± 1.21	4.47 ± 1.21	0.102
Fruit	4.07 ± 1.53	4.05 ± 1.54	4.27 ± 1.43	< 0.001
Cereal	2.691 ± 1.09	2.690 ± 1.09	2.70 ± 1.11	0.791
Fiber	4.86 ± 0.43	4.86 ± 0.43	4.90 ± 0.36	0.029
Protein	4.69 ± 0.25	4.96 ± 0.26	4.99 ± 0.11	0.008
Iron	4.80 ± 0.58	4.79 ± 0.60	4.86 ± 0.48	0.003
Calcium	2.57 ± 1.47	2.56 ± 1.47	2.61 ± 1.45	0.487
Vitamin C	4.95 ± 0.41	4.59 ± 0.42	4.96 ± 0.37	0.711
Moderation	11.92 ± 5.54	11.79 ± 5.47	13.27 ± 6.05	< 0.001
Total fat	2.52 ± 1.79	2.54 ± 1.79	2.38 ± 1.77	0.036
Saturated fat	2.62 ± 1.37	2.61 ± 1.37	2.72 ± 1.37	0.046
Cholesterol	3.72 ± 2.57	3.72 ± 2.57	3.80 ± 2.57	0.453
Sodium	2.32 ± 2.29	2.30 ± 2.28	2.58 ± 2.38	0.003
Empty foods	0.71 ± 1.51	0.61 ± 1.38	1.77 ± 2.28	< 0.001
Overall balance	2.86 ± 2.20	2.88 ± 2.21	2.61 ± 2.13	0.004
Macronutrients ratio	2.14 ± 1.88	2.15 ± 1.88	2.04 ± 1.84	0.176
Fatty acid ratio	0.71 ± 1.28	0.72 ± 1.29	0.56 ± 1.17	0.003

The scores of DQI-I subgroups are presented as (mean ± standard deviation). The p-values are obtained from t-test.

Logistic regression analysis was performed to compare the total DQI-I score between healthy groups and patients with diabetes. In model 1, the odds ratio (OR) for diabetes increased by 4% (OR  1.04; 95% CI 1.02–1.04). In model 2, after adjusting for confounding factors, the OR for diabetes increased by 53% (OR  1.53; 95% CI 1.34–1.75). The odds ratio in Model 3 decreased by 2% compared to Model 2. Therefore, Model 3 showed that the odds ratio for diabetes was 49% higher (OR  1.49; 95% CI 1.302–1.730), and all these values were statistically significant (Table 5).

**Table 5:** Association between DQI-I and diabetes using binary logistic regression.

Models	Odds ratio	95% confidence interval	*P*-value
Model 1	1.04	(1.027–1.049)	< 0.001
Model 2	1.53	(1.341–1.757)	< 0.001
Model 3	1.49	(1.302–1.730)	< 0.001

Model 1: Crude model, Model 2: Adjusted for age, gender, smoking status, and alcohol consumption. Model 3: Adjusted for Model 2 and marital status, energy, BMI, physical activity, supplements and socio-economic status.

The results show that the frequency of healthy individuals in the first tertile of total DQI-I was higher than in the other tertiles, which means that the healthy individuals were less adherent to their diet (37.77% in the first tertile vs. 29.08% in the third tertile). On the contrary, the frequency of diabetics in the third tertile was higher than in the first and second tertile, meaning that they adhered more to the dietary guidelines (29.44% in the first tertile vs. 42.76% in the third tertile). The DQI-I score of men was higher than the DQI-I score of women (*P* < 0.001). BMI, waist circumference (WC), systolic and diastolic blood pressure significantly increased from the first tertile to the second tertile (*P* < 0.05). Fasting blood sugar decreased from the first tertile to the second tertile (95.83 ± 27.35 mg/dl vs. 95.31 ± 27.03 mg/dl), but increased from the second tertile to the third tertile (100.83 ± 35.89 mg/dl) (*P* < 0.001) (Table 6).

**Table 6:** Frequency and distribution of different variables by DQI-I tertile.

Variable		All	DQI-I tertiles			*P*-value
			Tertile 1 Cut point < 33.33	Tertile 2 66.66 > Cut point < 33.33	Tertile 3 Cut point > 66.66	
Number	–	7115	2635 (37.03)	2327 (32.71)	2153 (30.26)	–
Age	Year	47.14 ± 8.39	47.54 ± 8.39	46.97 ± 8.29	46.83 ± 8.19	0.007
Gender	Men	3160	977 (30.92)	1067 (33.77)	1116 (35.32)	< 0.001
	Women	3955	1658 (41.92)	1260 (31.68)	1037 (26.22)	
Residence	City	4114	1197 (29.10)	13.81 (31.57)	1536 (34.37)	< 0.001
	Rural	3001	1436 (47.92)	946 (31.52)	617 (20.56)	
Socioeconomic status	Poor	1420	570 (40.14)	490 (34.51)	360 (25.35)	< 0.001
	Middle	1408	495 (35.16)	472 (33.52)	441 (31.32)	
	Rich	1375	416 (30.25)	471 (34.25)	448 (35.49)	
Physical activity	Low	2112	236 (38.19)	704 (33.33)	717 (33.95)	< 0.001
	Moderate	3481	1348 (38.72)	1074 (30.85)	1059 (30.42)	
	Vigorous	1522	596 (39.16)	549 (36.07)	377 (24.77)	
Body mass index	kg/m^**2**^	27.41 ± 4.66	27.08 ± 4.80	27.47 ± 4.63	27.75 ± 4.47	< 0.001
Waist circumference	cm	97.06 ± 10.59	96.32 ± 10.97	97.08 ± 10.61	97.92 ± 10.03	< 0.001
Systolic blood pressure	mmHg	107.65 ± 16.76	106.58 ± 16.66	107.69 ± 16.67	108.93 ± 16.90	< 0.001
Diastolic blood pressure	mmHg	69.44 ± 9.79	69.06 ± 9.66	69.34 ± 9.66	70.00 ± 10.06	0.003
Fast blood sugar	mg/dl	97.18 ± 30.19	95.83 ± 27.35	95.31 ± 27.03	100.83 ± 35.89	< 0.001
Diabetes	No	6471	2444 (37.77)	2145 (33.15)	2882 (29.08)	< 0.001
	Yes	608	179 (29.44)	169 (27.80)	260 (42.76)	

Variables are reported as frequency (%). For p-values, chi-square has been used to evaluate the distribution of classified variables and DQI-I tertile. Quantitative variables are reported with mean ± standard deviation. The p-values of ANOVA analysis is used to evaluate the differences of continuous variables in the tertiles of the DQI-I.

## Discussion

The results of the study showed that diet and food quality play an important role in the development of diabetes. Diabetics had a higher dietary quality than the healthy group and it had a significant influence on the overall balance. The main reason for this may be that diabetics follow a guideline-appropriate diet and their diet has been modified over the years. Safari Faramani et al. showed that most diabetics eat a healthy diet. Diabetics are affected by the behavioral changes after diabetes and tend to follow a healthy diet^18^.

In a Chinese study, a mean DQI-I score of 53 ± 0.75 was found for diabetics^19^. Furthermore, no association between variety subgroup and diabetes was found in this study, which is consistent with another study by Danquah et al. (2018)^20^. It is important to note that greater dietary variety may also lead to higher energy intake^21–23^. Variety has been found to have a positive association with energy availability at the family level in both urban and rural areas and with overweight and obesity at the individual level^24^. In this study, although no association was found between the adequacy subgroup and diabetes, previous research has shown that eating fruit and vegetables rich in fiber can reduce the insulin response to carbohydrates^25^.

The results of this study suggest that diabetics in the overall balance subgroup had lower levels. Therapeutic nutrition plays a fundamental role in the treatment of diabetes. The guidelines emphasize that different dietary patterns focusing on macronutrient composition can be tailored to individual needs to improve glycemic control and cardiovascular risk factors^26,27^.

When comparing different diets to improve metabolic diseases, it is also clear that a balance between macronutrients and their quality is crucial in nutritional interventions^28^. Vitale et al. demonstrated that even small changes in the ratio of carbohydrates to fats in the diet were associated with significant changes in metabolic risk factors and low-grade inflammation in diabetic patients, independent of energy intake and the use of blood glucose-lowering medications^26^. Tay et al. found that a low-carbohydrate diet with a higher proportion of unsaturated fatty acids compared to saturated fatty acids improves daily stability of blood glucose levels and positive changes in lipid profiles, with sustained effects^29^.

It is important to note that a low-carbohydrate diet may limit the intake of high-fiber foods and carbohydrate sources from vegetables, whole grains and fruits. However, one study has shown that a low-carbohydrate diet with a higher proportion of unsaturated fatty acids compared to saturated fatty acids does not affect nutritional biomarkers^30^. Furthermore, studies have shown that consuming a high protein diet with low fiber content can lead to metabolic acid production and increased acid load, which in turn impairs insulin sensitivity^31^. Nevertheless, protein consumption has been shown to improve glycemic control^32^.

The other component of the subgroup analyzed in this study was the overall balance of fatty acid ratios. A diet rich in fatty acids and glucose can induce epigenetic changes that affect the transcription of key genes involved in lipid and glucose metabolic pathways and inflammation^33^. Saturated fatty acids and their derivatives are generally more involved in the activation of inflammation and the maturation of certain cytokines and interleukins in the inflammatory process^32^. Arbabi Jam et al. have shown a positive relationship between diabetes and its risk factors and a highly pro-inflammatory diet^34^. In addition, the consumption of PUFAs has been associated with an improvement in blood lipid profiles, while the replacement of SFAs with MUFAs has been shown to worsen insulin and glucose homeostasis^33^. The American Heart Association (AHA) recommends limiting the intake of SFAs and replacing them with PUFAs^35^. PUFAs are generally considered healthy fats and play a critical role in blood vessel function, cell membranes, inflammation and the nervous system. They also serve as precursors for eicosanoids and lipid mediators such as resolvins, docosanoids, and protectins^36^.

Since the study referred to the prevalence of diabetes, the lifestyle of diabetics has therefore changed over the years of having the disease. However, it is important to consider the composition of macronutrients and the consumption of unsaturated fatty acids. These dietary modifications contribute significantly to improving the overall quality of the diet of people with diabetes. Furthermore, improving the nutritional knowledge of diabetics not only benefits their well-being but also helps to reduce the burden of this disease on society as a whole.

## Conclusions

The diabetics in the Ravansar region had a higher total DQI-I score compared to the healthy population. This result suggests that diabetics may have adopted healthier eating habits after their diagnosis. However, it is important to recognize that these improvements in dietary habits may be influenced by lifestyle changes rather than the quality of the diet itself. The main challenge remains to achieve a balanced overall diet composition, particularly in terms of macronutrients and fatty acids. Although further studies are needed to confirm these findings in the Kurdish population, this study emphasizes the importance of continuous dietary management and education of diabetic patients. Future research should focus on longitudinal studies to better understand the impact of dietary changes on the incidence of diabetes.

## Data Availability

The data analyzed in the study are available from the corresponding author upon reasonable request.

## Abbreviations

DQI-I: Dietary quality index-international
DRI: Dietary reference intakes
IDF: International diabetes federation
FFQ: Food frequency questionnaire
MUFA: Monounsaturated fatty acid
RaNCD: Ravansar non-communicable diseases
PUFA: Polyunsaturated fatty acids
